# Evaluation of Ultrasonically ZnO Loading Effect on Photocatalytic Self-Cleaning, UV Protection and Antibacterial Activity of Plasma/Citric Acid-Activated Cotton Fabric

**DOI:** 10.3390/nano12122122

**Published:** 2022-06-20

**Authors:** Muhammad Irfan, Humaira Hussain, Bisma Saleem, Muhammad Saleem, Shazia Shukrullah, Stanislaw Legutko, Jana Petrů, Muhammad Yasin Naz, Marek Pagáč, Saifur Rahman, Rehan Khan

**Affiliations:** 1 Electrical Engineering Department, College of Engineering, Najran University Saudi Arabia, Najran 61441, Saudi Arabia; miditta@nu.edu.sa (M.I.); srrahman@nu.edu.sa (S.R.); 2 Department of Chemistry, University of Okara, Okara 56300, Pakistan; humaira0949@gmail.com; 3 Department of Physics, University of Agriculture Faisalabad, Faisalabad 38040, Pakistan; saleem261996@gmail.com (B.S.); saleem_192@yahoo.com (M.S.); 4 Faculty of Mechanical Engineering, Poznan University of Technology, 3 Piotrowo Street, 60-965 Poznan, Poland; stanislaw.legutko@put.poznan.pl; 5 Department of Machining, Assembly and Engineering Metrology, Mechanical Engineering Faculty, VŠB-Technical University of Ostrava, 17. Listopadu 2172/15, 708 00 Ostrava, Czech Republic; jana.petru@vsb.cz (J.P.); marek.pagac@vsb.cz (M.P.); 6 Department of Mechanical Engineering, College of Electrical and Mechanical Engineering, National University of Sciences and Technology, Islamabad 44000, Pakistan; mrehan.khan@ceme.nust.edu.pk

**Keywords:** cotton fabric, ZnO nanoparticles, DBD plasma, self-cleaning, UV protection, sonification process, antibacterial activity

## Abstract

Zinc oxide (ZnO) nanoparticles were loaded over non-thermal plasma (P1) and citric acid (P2)-functionalized cotton fabrics using a room temperature sonification process. The cotton samples were pretreated with dielectric barrier discharge (DBD) plasma and citric acid to introduce some reactive moieties on the fabric to enhance the adhesion power of ZnO nanoparticles with an average particle size of 41 nm. The nanoparticles were dispersed homogeneously on the surface of the P1 sample, which enhanced the antibacterial, UV protection and photocatalytic self-cleaning characteristics of ZnO-loaded fabric. The self-cleaning efficiency of P1 and P2 samples was measured to be about 77% and 63%, respectively. The inhibition zones of 5.5 mm and 5.4 mm were produced by sample P1 against *E. coli* and *S. aureus* *bacteria*, respectively, which were slightly higher than the inhibition zones produced by sample P2. The inhibition zone of the samples roughly decreased by 17% after performing 10 wash cycles. The unloaded cotton fabric had a UPF value of 70.02 units and blocking percentage of 70.92% and 76.54% for UVA and UVB radiations, respectively. The UVA-blocking capacity of samples P1 and P2 was 95.27% and 91.22, respectively. Similarly, the UVB blocking capacity was 94.11% and 92.65%, respectively. The pre-coating plasma treatment was found to be helpful in improving the UV-blocking ability of ZnO-loaded cotton fabric.

## 1. Introduction

The fast-rising environmental pollution index of South Asian countries is putting human health at serious risk. Pollution occurs when dangerous chemicals are released into certain sections of the ecosystem. This could be the ash formed by a naturally erupted volcano, or it could be harmful fumes released through the combustion of fossil fuels. It is difficult to simultaneously treat the environment for all types of pollutants [1]. Pakistan is among the top garment-manufacturing and exporting countries. The garment sector contributes to 80% of the total exports of the country. In the textile processing industry, a number of physical and chemical processes are carried out for the finishing of fabrics [2,3]. About 98% of textiles are dyed at early or later stages before hitting the market. A considerable portion of the dye is lost to the wastewater stream during the coloring process because it does not adhere to the fabric [4]. The absorption and reflection of sunlight into aquatic bodies is a major environmental hazard caused by synthetic dyes. Because algae are at the bottom of the food chain, light absorption reduces their photosynthetic activity, which has major consequences for the food chain. One of the main reasons that aquatic life suffers in regions where dyes are dumped is a lack of algae. The synthetic dyes are also toxic themselves [5].

The self-cleaning and UV protection factors of fabrics are also among the rising human health concerns. UV radiation is primarily emitted by sunlight. UV radiations of different kinds reach the earth in different amounts. UVA photons account for approximately 95% of UV rays that reach the earth, with UVB photons accounting for the remaining 5%. UV exposure can cause premature skin ageing and sun damage signs such as leathery skin, liver spots, wrinkles, actinic keratosis and solar elastosis [6]. UV radiations can also cause corneal inflammation or burns, as well as the production of cataracts and pterygium, both of which can impair vision. Some people are more vulnerable to the harmful effects of UV rays. Some drugs can make people more sensitive to UV rays, increasing their chances of being burnt under sunlight [7]. The goal of textile finishing is to shield the skin from the effects of sun radiation because the textile does not always provide appropriate protection. Textiles’ specific protective properties against a wide range of impacts have gained increasing attention. Unfinished clothes have a limited light retarding capacity that prevents them from providing suitable protection to the human body from UV radiation [3]. As a result, UV stabilizers or protective coatings are used to offer an extra layer of sun protection. Textiles’ UV protection factor is determined by their architecture, yarn spacing, fiber type, textile impregnation, color, and the presence of UV absorbers and optical brighteners. The ultraviolet protection factor (UPF) of natural fibers is lower than that of synthetic fibers. Among natural fibers, cotton fabric in a grey state has a greater UPF due to natural colors, pectins, and waxes. Undyed and bleached cotton fabrics have a low UPF, while dyed cotton fabrics have a high UPF [8]. UV absorbers are organic or inorganic substances that show strong absorption in the wavelength range of 290–360. Electronic excitation energy is converted to thermal energy via UV absorbers embedded within the fibers. They act as oxygen scavengers and radical scavengers [9]. High-intensity UV rays stimulate the UV absorber, causing it to absorb a large amount of energy, which is then dispersed as longer-wave radiation. Isomerization, on the other hand, can occur, causing UV absorbers to fragment into non-absorbing isomers. 

This study focused on the protective coating of metal nanoparticles on the fabric surface to impart self-cleaning and UV blocking traits. A green synthesis method, coupled with the sonification process, was considered for the concurrent synthesis of nanoparticles and coatings over fabric. Green synthesis is an effective and eco-friendly way of producing nanomaterials that offer economic and environmental friendly solutions compared to chemical and physical methods [10]. Green synthesis is a simple, low-cost, and environmentally friendly process that does not require high temperature, pressure, energy, and harmful chemicals. The bioactive products of plants, bacteria, fungi and yeasts can be an excellent source for the synthesis of nanoparticles. In this study, we synthesized metal oxides of zinc with a green method using the extract of *Azadirachta indica* and studied their photocatalytic [11], UV protection and antibacterial activities by coating them onto an atmospheric pressure plasma-treated cotton fabric. *Azadirachta indica,* also called Neem, is an indigenous tree, which belongs to the mahogany family Meliaceae. The medicinal benefits of *Azadirachta indica* are outstanding. *Azadirachta indica* leaves contain a variety of biologically active phytoconstituents, including flavonoids, alkaloids, terpenoids and polyphenols, which can be utilized to reduce metal ions into their nanoparticles [12].

With a band gap of 3.37 eV and a large exciton binding energy of 60 meV, ZnO is a good n-type semiconductor which can be used in biosensors, solar cells, gas sensors, light detectors, the textile industry and cosmetics [13]. Because of its comparable band gap value and low cost, ZnO is considered as an effective alternative photocatalyst for TiO_2._ Hence, nanoparticles of ZnO and TiO_2_ could be employed as the best photocatalytic material for the degradation of organic pollutants. In order to treat infections due to antibiotic-resistant bacteria, it is becoming increasingly vital to develop novel antibacterial nanomaterials [14]. Photocatalytic activity, antibacterial activity and other applications of ZnO nanoparticles make them important materials in the medical and energy industries. ZnO nanoparticles have been used by several researchers to derive photocatalytic reactions for the degradation of organic pollutants and compounds in both visible and UV regions of the electromagnetic spectrum. Photocatalysis occurs when a semiconductor photocatalyst is subjected to UV radiations, which excites the electrons and causes them to move to the conduction band by creating holes in the valence band [15,16]. These electrons and holes take part in redox reactions to degrade dye molecules and neutralize bacteria.

The adhesion and binding of nanoparticles remains an important issue in the textile industry. Nourbakhsh et al. [17] functionalized polyester fabric with NaOH to increase the stability and binding ability of fabric for ZnO. The chemical alkaline treatment of fabric can damage its structure and weakens the strength of the fiber. Additionally, it produces water pollution by discharging chemicals. The corrosive nature and high concentration of NaOH produce the bulk of wastewater, which causes the rinsing of treated textiles. Therefore, a dry, environmentally friendly and cost-effective approach is sought for the surface modification of fabric without damaging its bulk structure. The treatment of cotton with non-thermal plasma can improve its various properties. The surface properties of cotton can be improved by generating plasma in the presence of non-polymerizing gases. Atmospheric pressure plasma can remove non-cellular impurities from the surface of cotton through a physical etching process. Plasma treatment enhances hydrophilicity as well as sizing, dyeing, adhesion and surface roughness [2]. In plasma treatment, the surface of cotton fabric is incorporated with plasma-generated free radicals, which act as grafting or cross-linking agents. So, the activation of cotton with plasma results in better wettability, antistatic behavior, adhesion of non-coatings dyeing, printability, cleaning, disinfection of surface and electrical properties [18]. The main objective of this research was to find the best technique for enhancing the adhesion property of ZnO nanostructures on a cotton substrate. Two techniques were used to functionalize the cotton fabric. The first was to treat the cotton with citric acid binder and the second was to treat the cotton with dielectric barrier discharge plasma. 

## 2. Materials and Methods

### 2.1. Precursor Materials

Chemicals of an analytical reagent grade, including wetting agent, Citric acid (C_6_H_8_O_7_), Zinc nitrate (Zn(NO_3_)_2_·6H_2_O), sodium chloride, and sodium hydroxide were supplier of Merck (Burlington, VT, USA). The commercial-grade methylene blue was purchased from a local scientific store in Faisalabad, Pakistan. The unprocessed woven cotton fabric was procured from Sapphire Textile limited, Sheikhupura, Punjab, Pakistan. The woven fabric had 100 ends, 85 picks and a density of 118 g/m^2^. The raw fabric was desized to remove starch, dirt, and other contaminants before the coating tests.

### 2.2. Desizing of Cotton Samples

The woven cotton was cut into pieces of 10 × 10 cm^2^ dimensions and desized in hot distilled water with 2 g/L sodium chloride, 3.5 g/L enzyme and 2 g/L wetting agent. Desizing was conducted for 2 h. The desized samples were heat treated in an oven at 90 °C and stored for the plasma treatment and nanocoating of ZnO nanoparticles.

### 2.3. Preparation of Neem Extract

Green leaves of Neem plant with the scientific name “*Azadirachta indica*” were taken from a local botanical garden in Faisalabad, Pakistan. The fresh leaves were washed with DI water several times to remove dust particles and other impurities. The washed leaves were dried in an open environment for 7 days under shade. The leaves were then ground into a fine powder. About 20 g of Neem powder was taken in a glass beaker. About 150 mL of ethanol was added to the glass container, following which it was covered with aluminum foil and placed in a dark room overnight. After that, the solution was heated on a magnetic hotplate for 2 h at 70 °C under continuous stirring. The solution was then cooled and filtered to obtain the leaf extract for use in the green synthesis of ZnO.

### 2.4. Activation of Cotton with DBD Plasma and Citric Acid

**Figure 1:**
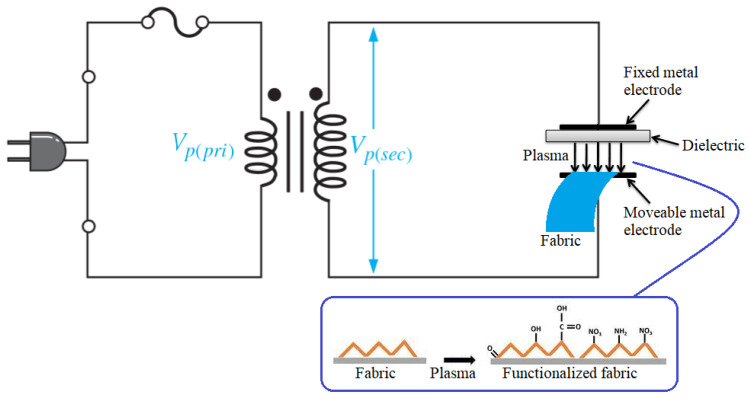
Illustration of DBD plasma system for activation of cotton fabric in open air.

A desized sample of cotton was pasted onto a movable electrode of the DBD plasma system, as illustrated in Figure 1. The fabric-carrying electrode was rotated at 100 rpm under plasma exposure. The plasma was generated between an interelectrode gap of 2 mm. The DBD microdischarge was generated in an open gap with air as the source gas. A schematic of the DBD plasma system for the functionalization of the cotton surface is shown in Figure 1. The operating conditions for the plasma produced were as follows: input power of 106 W, discharge current 3.8 mA and discharge voltage of 26 kV. The cotton sample was activated from both sides with plasma for the optimum exposure time of 70 s, as reported in our previous work [10].

In the pre-coating citric acid treatment, the cotton fabric was functionalized with 0.5% dilute solution of citric acid in order to increase the adhesion of ZnO particles. Equations (1) and (2) show that when the cotton fibers and citric acid are placed in deionized water, both become ionized. In processing reactions, the hydroxyl groups (OH) on cotton are attached with the carboxylic groups of acid using the following Equation (3) [19].

(1) $$H_{2}O+C_{6}H_{8}O_{7}↔C_{6}H_{7}O_{7}^{-}+H_{3}O^{+}$$

(2) $$\mathrm{Cellulose}-\mathrm{OH}+H_{2}O↔\mathrm{Cellulose}-O_{}^{-}+H_{3}O^{+}$$

(3) $$C_{6}H_{8}O_{7}+\mathrm{Cellulose}-\mathrm{OH}+H_{2}O↔\mathrm{Cellulose}-\mathrm{CA}+H_{3}O^{+}$$

### 2.5. Synthesis and Coating of ZnO Nanoparticles on Activated Fabric

The ZnO nanoparticles were synthesized using a green method. About 150 mL of Neem leaf extract was heated on a magnetic stirrer at 60 °C for 10 min. After that, 0.1 M solution of zinc nitrate (50 mL) and 0.2 M solution of sodium hydroxide (20 mL) were added to 100 mL of the extract in the stirring mode. NaOH is a basic reducing agent for metal salts. It reacts exothermically and often violently with oxidizing agents of all types. Neem extract was used because it is a low-cost active biological component that can function as a reducing, stabilizing, and capping agent. When coupled with NaOH, it expedites the reaction rate by cutting down the reaction time. Neem extract contains terpenoids and flavanones, which help in stabilizing nanoparticles. Here, a one-pot reaction, facile, safe and eco-friendly co-precipitation approach that utilized *Azadiratcha Indica* extract along with NaOH as the alkaline medium was performed [20]. Only 30 min was required for the complete conversion of metal ions into nanoparticles at room temperature. In the absence of neem extract, 2 h was required to complete the reaction. The mixture turned into cream-colored precipitates of ZnO after 30 min of continuous stirring. The precipitates were centrifugated at 3000 rpm and washed with distilled water. After drying in an oven, the power was ground to produce fine nanoparticles of ZnO. The nanoparticles were calcinated in an electric furnace for 1 h at 300 °C.

The DBD plasma-treated and citric acid-treated samples were placed in a 3% solution of ZnO in separate beakers for five minutes. The solutions and fabric were then sonicated for 15 min at 75 °C. Then, the samples were removed from the sonication bath, air-dried and cured at 140 °C. The samples were rinsed 5 times to wash out the impurities and unbounded nanoparticles. The pre-citric acid-treated ZnO-coated sample was named as P1 and the post-citric acid-treated ZnO-coated sample was named as P2. Finally, the produced samples were dried, characterized and used for antibacterial activity. The samples were also tested for UV protection and the removal of methylene blue from the solution exposed to UV light. 

UV-visible spectroscopy in the 300–700 nm region was used to investigate the optical characteristics of ZnO particles. XRD patterns of the P1 and P2 samples were obtained by using X-ray diffractometer Cu-Kα radiations in the range of 20° to 80°. The particle size, morphology and shape of ZnO coatings were analyzed by SEM. The attached functional groups during DBD plasma treatment were detected in the FTIR spectra in a series of absorbance peaks in 500–4500 cm^−1^ range.

### 2.6. Antibacterial and Self-Cleaning Activities of ZnO-Coated Samples

ZnO-coated samples were tested for antibacterial activity against *E. coli* and *S. aureus* strains. The well-diffusion method was used to determine the activity of ZnO against selected bacteria. Muller–Hinton agar medium was used to make the wells on plates. The plates were then seeded with two types of bacteria using a sterilized swab. Gel puncture was used to create four wells in each plate. Each well was filled with samples of ZnO-coated fabrics. To observe the zones of inhibition, the seeded plates were placed in an incubator at 35 °C for 24 h.

The degradation of methylene blue (MB) molecules under UV light exposure was studied using a 380 nm cutoff filter and a Xenon lamp. The P1 and P2 samples were added individually to 0.05% *w*/*v* MB dye solution to test their photocatalytic activity. The solutions with P1 and P2 samples were kept in the dark to produce the adsorption–desorption equilibrium between the dye and the coated sample. After that, the P1-containing solution was irradiated with UV light for different time intervals. For different hours of the irradiation of UV light, the UV-visible spectra of the samples were recorded. The absorption of light by MB solution was observed at 665 nm and its UV–Visible absorption spectra were recorded to estimate the rate of dye breakdown. The Beer–Lambert law (A = ε_m_CL) was used to calculate the dye concentration in the solution. The photocatalytic efficiency of ZnO-coated cotton fabric towards MB was determined using the following formula:

(4) $$\mathrm{Degradation}\;\mathrm{efficiency}\;(\%)\;=\;\left[1-\frac{C}{C_{o}}\right]\times 100$$

where, C_o_ is the initial dye concentration in solution and C is final dye concentration. Similarly, the photocatalytic efficiency of the P2 sample was also measured.

### 2.7. Ultraviolet Protection

The Varian CARY UV-Vis instrument (Agilent Technologies, Santa Clara, CA, USA) was employed to investigate the UV protection ability of the developed fabric samples. A spectrophotometer consisting of solar screen software and an integrating sphere with a wavelength range of 280–400 nm was operated under a standard AATCC 183–2000. Using this standard, the percentage of transmission and blocking of UV radiation through the fabric samples were determined. The samples were handled at standard temperature conditions (25 ± 2 °C) and a relative humidity of 66 ± 2%. The average value of the five measurements was taken. The following Equation (5) was used to determine UPF [21]:

(5) $$\mathrm{UPF}=\frac{\sum_{280\;\mathrm{nm}}^{400\;\mathrm{nm}}E_{\lambda }S_{\lambda }{∆}_{\lambda }}{\sum_{280\;\mathrm{nm}}^{400\;\mathrm{nm}}E_{\lambda }S_{\lambda }T_{\lambda }{∆}_{\lambda }}$$

where, $E_{\lambda }$ is the solar irradiance, $S_{\lambda }$ spectral response of relative erythemal, ${∆}_{\lambda }$ is wavelength in nm and $T_{\lambda }$ is percentage transmittance.

## 3. Results and Discussion

### 3.1. FTIR Analysis of DBD Plasma Treated Cotton

**Figure 2:**
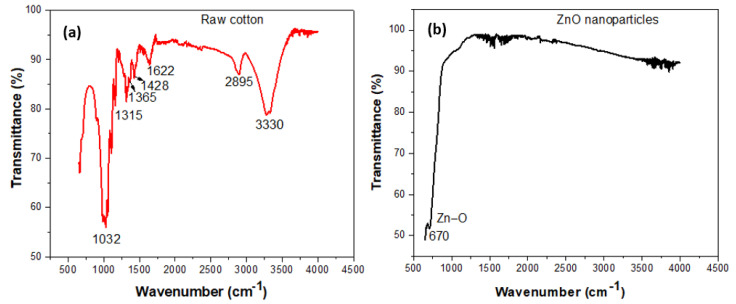
FTIR spectra of (**a**) raw cotton and (**b**) as-produced ZnO nanoparticles.

FTIR spectra (Agilent Technologies, Santa Clara, CA, USA) of raw cotton, and as-produced ZnO nanoparticles are reported in Figure 2. The FTIR spectrum in Figure 2a revealed the hemicellulose, lignin and cellulosic bands in the spectrum of raw cotton. The band at 3330 cm^−1^ corresponds to a characteristic peak of cellulosic hydroxyl groups, water and lignin. A partially strong peak at 2895 cm^−1^ corresponds to the stretching vibration of cellulosic C-H. The presence of water in cotton fabric corresponds to the 1622 cm^−1^ band. The band at 1315 cm^−1^ corresponds to the bending vibrational mode of the hydrocarbon structure. The bands at 1365 cm^−1^ and 1428 cm^−1^ refer to the cellulosic molecule’s originated symmetric stretching of carboxylates (CH_2_ and C-H). A strong peak at the 1032 cm^−1^ band appeared due to the stretching vibration of the polysaccharide of cellulose. On the other hand, the FTIR spectrum in Figure 2b shows a band between 500 cm^−1^ and 1000 cm^−1^, which is the characteristic mode of ZnO nanoparticles. The Zn-O bending vibration was confirmed by a medium band at 670 cm^−1^ [22]. 

### 3.2. UV-Vis Analysis of ZnO Nanoparticles

**Figure 3:**
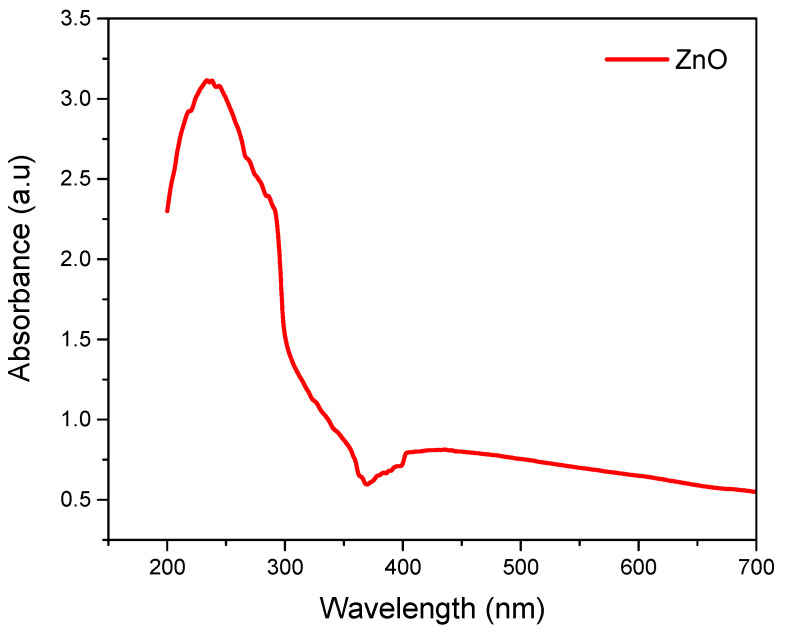
UV-vis profile of as-produced ZnO nanoparticles before coating over cotton fabric.

Varian carry 500 was used to obtain the UV-visible spectrum of the solutions containing ZnO nanoparticles, as shown in Figure 3. The peaks in the spectrum are the result of surface plasmon resonance of nanoparticles [23]. The electron’s collective excitation in the conduction band close to the surface of the nanoparticles is called surface plasmon resonance. At 250 nm, a prominent UV light absorption peak can be noticed. The Tauc-Plot method was used to discern the band gap energy of ZnO nanoparticles. The mathematic form of the Tauc relation is [24]:

(6) $$\alpha h\upsilon =A{\left(h\upsilon -E_{g}\right)}^{n}$$

where, *E_g_*, is the band gap, hυ is the energy of incident photons, α is the absorption coefficient, and *A* is the transition probability constant. The constant *n* can be ½ or 2 based on direct or indirect transitions. The energy band gap lies between 3.35 eV to 3.4 eV. It is worth noting that the perceived blue shift absorption is different from that of bulk ZnO, which has an absorption peak at around 355 nm due to the quantum confinement effect of ZnO nanostructures. The peaks change within the range of 250 nm to 350 nm. So, it was confirmed that the Neem leaves contain all the stabilizing and reducing agents essential for the synthesis of nanoparticles. When Neem extract was added to the solution, the solution turned to cream-colored precipitates in just 30 min compared to the solution containing NaOH. A fast change in color indicated the formation of ZnO nanoparticles due to the reduction of zinc salt. The rapid reduction of Zinc salt into nanoparticles can be attributed to terpenoids and flavanones compounds in the Neem extract [25]. A peak around 250 nm was due to the excitation of surface plasmon vibrations in ZnO nanoparticles.

### 3.3. XRD Analysis of ZnO-Coated Cotton

**Figure 4:**
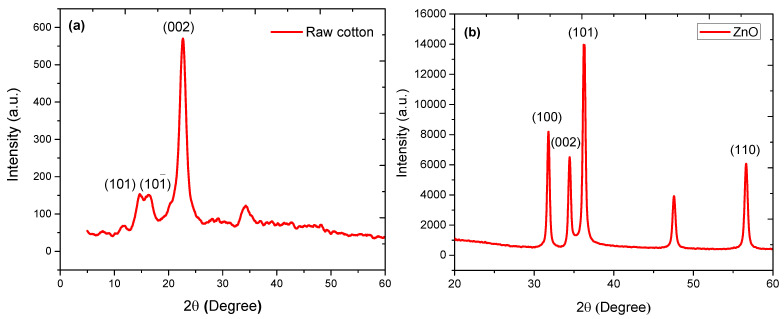
XRD spectra of (**a**) raw cotton and (**b**) as-produced ZnO nanoparticles before coating over cotton fabric.

The suitable method to study the crystalline nature of coated nanoparticles is X-rays diffraction. XRD patterns of blank cotton and ZnO nanoparticles, taken at a current of 20 mA and voltage of 40 kV in the 2 θ range of 0° to 80°, are shown in Figure 4. Figure 4a confirms the crystalline nature of the cellulose in cotton. A major XRD peak at 2θ of 22.6° is the characteristic peak of cellulose, describes the (002) crystalline plane. Some additional XRD peaks at 2θ of 14.9° and 16.5° correspond to the (101) crystalline plane. Figure 4b, on the other hand, revealed the hexagonal phase of as-produced ZnO nanoparticles. Some prominent XRD peaks were observed at 2θ of 31.54°, 34.40°, 36.71°, 47.45°, 56.36°, and 62.82°, corresponding to (100), (002), (101), (102), (110), (103) planes, respectively [26]. The intensity of the characteristic peak at 2θ of 34.40° was sharp as compared to other peaks in the (101) crystalline plane. A similar plane was observed in the crystalline structure of cellulose. The crystallite size of nanoparticles was measured by considering the full width at half maximum (FWHM) of the selected XRD peaks. The lower FWHM shows a large crystallite size. In contrast, large FWHM denotes a smaller crystallite size. The Scherrer equation was used to determine the size of crystallites.

### 3.4. SEM Analysis of ZnO-Coated Cotton 

**Figure 5:**
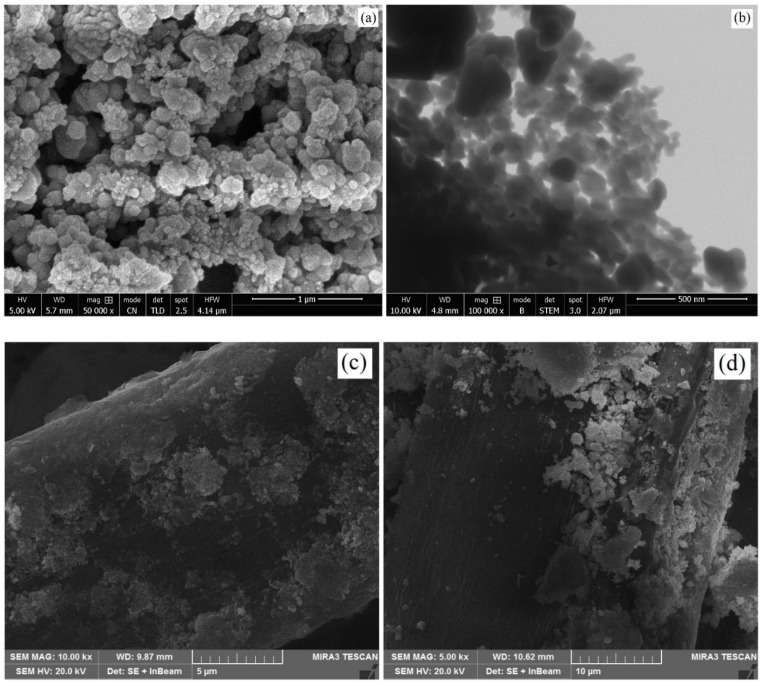
(**a**) SEM micrograph of as-produced ZnO, (**b**) STEM micrograph of as-produced ZnO, (**c**) SEM micrograph of ZnO-coated P1 sample and (**d**) SEM micrograph of ZnO-coated P2 sample.

The surface morphology of ZnO nanoparticles and coated cotton samples was analyzed by generating SEM and STEM images, as shown in Figure 5. The size and morphology of ZnO nanostructures are outlined in Figure 5a, which shows almost a spherical shape of the nanoparticles. The average particle size was measured to be about 40 nm. Figure 5b shows the STEM image of the nanoparticles. The STEM image reveals the low agglomeration of the nanoparticles. The dispersed nanoparticles make a strong bond with the fabric surface and show high stability over multiple wash cycles. Figure 5c,d reveal the SEM morphology of the ZnO-coated cotton samples (P1 and P2). ZnO nanoparticles dispersed homogeneously on the surface of plasma functionalized fabric (P1), which promoted the antibacterial and photocatalytic characteristics of the coated fabric. The coating on sample P2, on the other hand, was dense in some places. The nanoparticles in the dense parts did not make strong contact with the surface and showed slightly lower stability over multiple wash cycles compared to the plasma functionalized sample. For the plasma-treated sample, P1, the quantity of ZnO coating was higher than the citric acid-treated P2 sample. This was due to the fact that plasma induced some important moieties (COOH, NO_3_, NH_2_, O, OH) on the surface of cotton. These groups made a strong interaction with the nanoparticles. The occurrence of acoustic cavitation during the ultrasonic-assisted coating procedure produces shortly lived localized hot zones. The high temperature and pressure zones cause the sonolysis of water by producing hydrogen and hydroxyl radicals. The air trapped by the fabric turns into bubbles in the fabric. The transient bubbles cause powerful convection when they move near the fabric surface. This phenomenon intensifies the transfer and adsorption of nanoparticles to the fabric surface. The nanoparticles also revealed a strong affinity to the hydroxyl radicals produced during sonolysis of water. The hydroxyl radicals form strong interfacial bonding for the firm interaction of nanoparticles with the fabric surface.

### 3.5. Photocatalytic Activity

**Figure 6:**
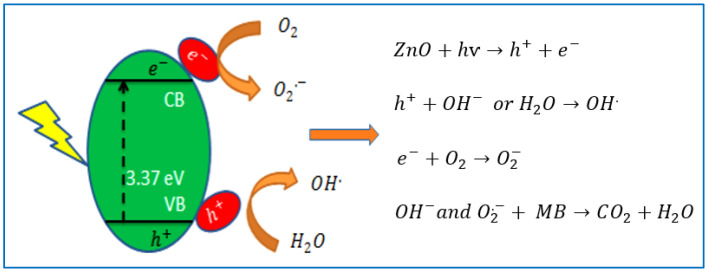
Mechanism of photocatalytic self-cleaning action of ZnO nanoparticles.

The photocatalytic action of ZnO nanoparticles, coated on samples P1 and P2, was assessed by measuring the intensity of the UV-vis spectra of the MB solution after administering UV light exposure for different intervals of time. The mechanism of the photocatalytic self-cleaning action of a coated fabric involves the photoexcitation of the ZnO photocatalyst and production of electron-hole pairs by the migration of electrons into the conduction band from the valance band [27]. The electrons and holes derive the redox reactions at the coated surface to degrade dye molecules, as illustrated in Figure 6.

**Figure 7:**
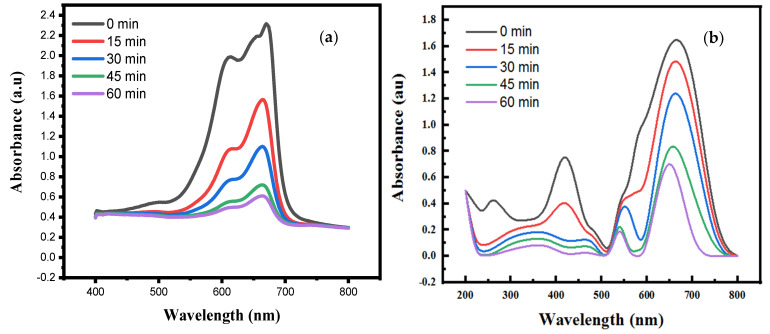
UV-vis profiles of dye solution after different UV exposure times in the presence of (**a**) coated fabric sample P1 and (**b**) coated fabric sample P2.

**Figure 8:**
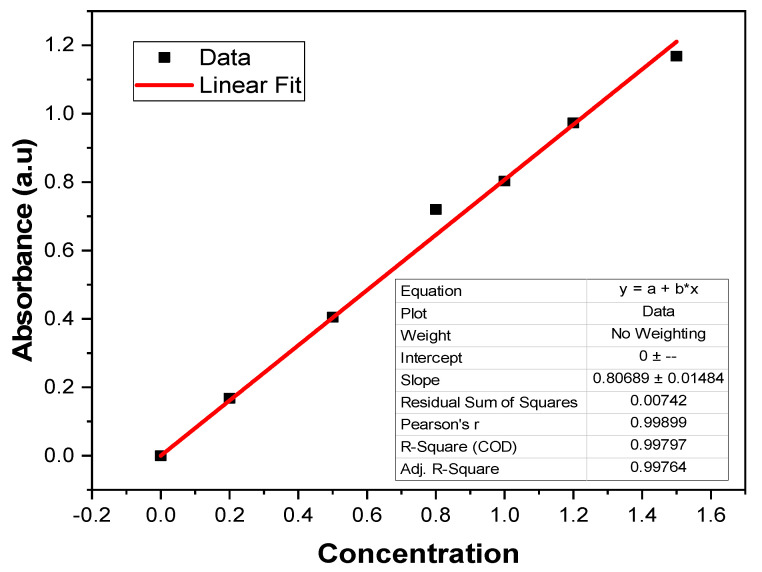
Linear fit of light absorbance by methylene blue dye solution.

Figure 7 shows UV-vis spectra of the dye-containing solution after UV irradiation for different time periods in the presence of ZnO-coated P1 and P2 samples. The concentration of dye decreased with the UV exposure time. The molar absorptivity of the dye solution was measured by drawing a linear fit of the absorbance of dye against a range of dye concentration, as shown in Figure 8. Dye degradation increases with the exposure time, which was confirmed by a decrease in light absorption by the solution. The sample P1 showed better self-cleaning activity than sample P2. The self-cleaning efficiency was 77% for sample P1 and 63% for sample P2. The dye molecules were degraded by following the above-mentioned mechanism and this suggested that the plasma-treated and ZnO-coated cotton has a high ability to degrade dye molecules [28]. 

### 3.6. Coating Stability Test

The weight of the control sample was estimated to be around 272.6 gsm, while the weight of ZnO-coated P1 and P2 samples was estimated to be around 283.1 gsm and 276.4 gsm, respectively. The nanoparticles interact with the fabric both physically and chemically by filling the voids between fibers and developing conductive networks throughout the fabric. A transparent tape was used to perform an adhesive test prior to the washing test. Some of the nanoparticles from the control sample bonded to the tape, indicating that nanoparticles have a weaker interaction with the fabric surface. Nanoparticles did not bind to the tape from P1 and P2 samples, indicating that the particles were tightly bonded to the fabric surface. The samples were then washed for 15 min according to ISO105–CO1 standard to verify the stability of nanoparticles on the fabric surface. Each test was repeated thrice and average values are reported in Table 1. Upon washing, the electrical resistivity of the control increased from 149 to 174 mm, showing that a substantial amount of nanoparticles was removed from the fabric surface. The samples P1 and P2 exhibited a slight increase in electrical resistivity from 122 Ω mm to 126 Ω mm and 127 Ω mm to 137 Ω-mm, respectively. A small change in electrical resistivity revealed the high stability of ZnO on the fabric surface, especially for the P1 sample. These results were further verified from the weight loss measurements. The weight loss in the control sample was 35.3 gsm, which was significantly larger than the weight loss in samples P1 and P2. The plasma treatment of P1 created pits and functional groups on its surface. As a result, ZnO loading increased due to pits filling and forming bonds between nanoparticles and plasma-induced functional groups.

### 3.7. Antibacterial Properties

**Figure 9:**
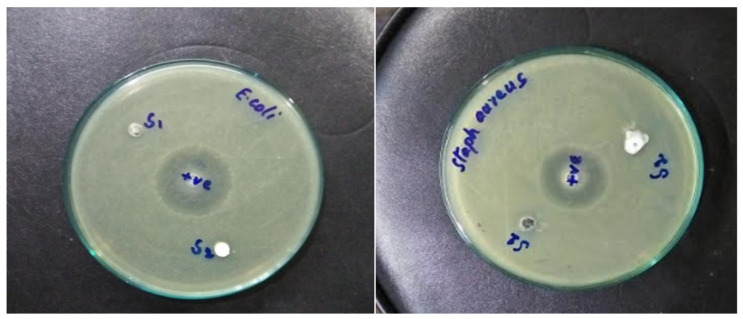
Photographic view of inhibition zones of P1 (**left**) and P2 (**right**) samples against *E. coli* and *S. aureus bacteria*.

Recently, different antibacterial agents were used to improve bacterial inhibition growth on cotton fabrics. Different nanomaterials such as TiO_2_ and silver nanoparticles were used as emerging antibacterial agents with unique antiviral properties. Figure 9 shows the inhibition zone of the ZnO-coated fabric against *E. coli* and *S. aureus* for samples P1 and P2. Clear inhibition zones of bacterial growth were noticed all around the ZnO-coated fabrics. Before washing, the coated fabrics retained more ZnO with a greater inhibition zone and showed larger bacterial reduction abilities. The inhibition zone of sample P1 was larger than P2 even after 10 washing cycles because greater quantities of ZnO attached to the fabric surface. Hence, plasma treatment enhanced the coating strength of fabric by inducing more functional groups. These results were obtained due to the biocidal action of ZnO-coated fabrics. Table 2 summarizes the inhibition zones of samples P1 and P2 against *E. coli* and *S. aureus bacteria*.

The sample P1 produced an inhibition zone of 5.5 mm and 5.4 mm against *E. coli* and *S. aureus bacteria*, respectively. Similarly, sample P2 produced an inhibition zone of 5.2 mm and 5.3 mm against *E. coli* and *S. aureus bacteria*, respectively. The inhibition zone of the samples roughly decreased by 17% after performing 10 wash cycles. This result indicates that some of the loosely bonded nanoparticles were washed away during multiple washing cycles. Overall, sample P1 showed superior antibacterial activity as compared to sample P1.

### 3.8. Ultraviolet Protection Factor

**Figure 10:**
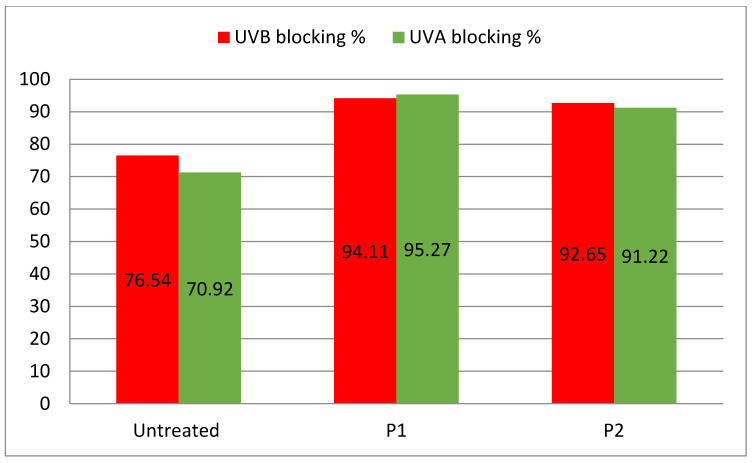
UVA and UVB blocking percentage values of unloaded and ZnO loaded P1 and P2 samples.

Sun radiation consists of three types of ultraviolet radiation, UVA with a wavelength range of 315–400 nm, UVB with a wavelength range of 280–315 nm and UVC with a wavelength range of 100–280 nm [14]. For the most part, UVC does not reach the earth because it is absorbed by the ozone layer of our atmosphere. UVA radiation is assumed to be dangerous for human skin since it causes sunburn, DNA modification, acne and skin cancer. UV protection provides the skin with the shielding ability of fabric against harmful radiation. The percentage of UV transmission through the fabric is described by the UPF value. The locking capacity of ZnO-loaded cotton fabric against UVA and UVB radiations is reported in Figure 10. The unloaded cotton fabric showed a UPF value of 70.02 units and its blocking percentage values for UVA and UVB were 70.92% and 76.54%, respectively. The UPF along with UVA and UVB blocking increased for the ZnO-loaded cotton samples. The UPF values of samples P1 and P2 were 95.27 and 91.22, respectively, which were much higher than the UPF of unloaded fabric. The UVA blocking values of samples P1 and P2 were 95.27% and 91.22, respectively. Similarly, UVB blocking values were 94.11% and 92.65%, respectively. These results showed that the pre-coating plasma treatment of fabric is slightly helpful in improving the blocking ability of ZnO-loaded fabric.

## 4. Conclusions

This research reveals that ZnO nanoparticles can be prepared from the extract of Neem plant leaves. This research also showed that zinc nitrate could be reduced by Neem leaves to prepare stable ZnO nanoparticles. The FTIR analysis confirmed the presence of functional groups of cellulose in raw fabric and ZnO coating on cotton fabric. Stronger and sharper diffraction characteristic peaks in the XRD pattern revealed a hexagonal ZnO phase with an average size of 41 nm. There was a dense coating of ZnO on the surface of the P1 sample, which was subjected to DBD plasma treatment. DBD plasma treatment increased the coating quantity and uniformity of nanoparticles as compared to citric acid treatment. The ZnO-loaded cotton fabric showed antibacterial properties against *E. coli* and *S. aureus bacteria*. The P1 sample showed greater antibacterial capability than the P2 sample due to more ZnO coating on its surface even after 10 washing cycles. The developed ZnO-loaded P1 and P2 samples were also used to degrade the methylene blue dye. The reduction in absorbance by the dye solution in the presence of coated cotton was 77% and 63% for samples P1 and P2, respectively. The plasma treatment increased the induction of important functional groups, which in turn increased the density of loaded ZnO nanoparticles and enhanced the photocatalytic efficiency. The UPF value for the P1 sample was much higher than the blank sample. The UPF value of the plasma-treated sample was also slightly higher than the P2 sample. Overall, the biosynthesized ZnO nanoparticles exhibited an excellent antimicrobial ability and proved to be a better photocatalyst for the removal of organic pollutants from wastewater and self-cleaning applications. So, the plasma treatment method proves to be a feasible method for the dense loading of nanoparticles on textiles for diverse applications.

## Figures and Tables

**Table 1:** Electrical resistivity and weight loss of ZnO-coated cotton.

Sample	Electrical Resistivity (Ω-mm)		Fabric Weight (gsm)	
	Before Washing	After Washing	Before Washing	After Washing
Control	149	174	272.6	237.3
P1	122	126	283.1	277.9
P2	127	137	276.4	259.3

**Table 2:** Inhibition zones of samples P1 and P2 against *E. coli* and *S. aureus bacteria*.

Washing Cycles	*E. coli*		*S. aureus*	
	P1	P2	P1	P2
0	5.5 mm	5.2 mm	5.4 mm	5.3 mm
10	4.6 mm	4.2 mm	4.5 mm	4.4 mm

## Data Availability

The reported data is available from the corresponding authors on valid request.
